# Saxagliptin Attenuates Albuminuria by Inhibiting Podocyte Epithelial- to-Mesenchymal Transition via SDF-1α in Diabetic Nephropathy

**DOI:** 10.3389/fphar.2017.00780

**Published:** 2017-11-01

**Authors:** Yun-peng Chang, Bei Sun, Zhe Han, Fei Han, Shao-lan Hu, Xiao-yu Li, Mei Xue, Yang Yang, Li Chen, Chun-jun Li, Li-ming Chen

**Affiliations:** Key Laboratory of Hormones and Development (Ministry of Health), Tianjin Key Laboratory of Metabolic Diseases, Tianjin Metabolic Diseases Hospital and Tianjin Institute of Endocrinology, Tianjin Medical University, Tianjin, China

**Keywords:** diabetic nephropathy, podocyte epithelial-to-mesenchymal transition, saxagliptin, NOX2, SDF-1α

## Abstract

The dipeptidyl peptidase-4 (DPP-4) inhibitor saxagliptin has been found to reduce progressive albuminuria, but the exact mechanism of inhibition is unclear. Podocyte epithelial-to-mesenchymal transition (EMT) has emerged as a potential pathway leading to proteinuria in diabetic nephropathy (DN). Stromal cell–derived factor-1α (SDF-1α), one of the substrates of DPP-4, can activate the protein kinase A pathway and subsequently inhibit its downstream effector, transforming growth factor-β1 (TGF-β1), which induces podocyte EMT. Thus, this study was designed to test the hypothesis that saxagliptin reduces progressive albuminuria by preventing podocyte EMT through inhibition of SDF-1α cleavage in DN. The results of a series of assays, including ELISA, western blotting, and immunochemistry/immunofluorescence, showed that saxagliptin treatment obviously ameliorated urinary microalbumin excretion and renal histological changes in high-fat diet/streptozotocin-induced diabetic rats. Furthermore, saxagliptin-treated diabetic rats presented with suppression of DPP-4 activity/protein expression accompanied by restoration of SDF-1α levels, which subsequently hindered NOX2 expression and podocyte EMT. *In vitro*, we consistently observed that saxagliptin significantly inhibited increased DPP-4 activity/expression, oxidative stress and podocyte EMT. Application of an SDF-1α receptor inhibitor (AMD3100) to cultured podocytes further confirmed the essential role of SDF-1α in podocyte EMT inhibition. In sum, we demonstrated for the first time that saxagliptin treatment plays an essential role in ameliorating progressive DN by preventing podocyte EMT through a SDF-1α-related pathway, suggesting that saxagliptin could offer renoprotection and that SDF-1α might be a potential therapeutic target for DN.

## Introduction

Diabetic nephropathy (DN) has become the leading cause of end-stage renal disease worldwide (Ritz et al., 1999). A recent study indicated that the population of people with chronic kidney disease related to diabetes in China was increasing, and the estimated number was 24.3 million (Zhang et al., 2016). Another reported cross-sectional study confirmed that the prevalence of diabetic kidney disease (DKD) in a total of 3301 patients with type 2 diabetes (T2DM) was 12.03% (Guo et al., 2016). Glucose-lowering strategies are considered the main way to preserve renal function, and several milestone trials, such as the Diabetes Control and Complications Trial (de Boer et al., 2011) and the Action in Diabetes and Vascular Disease: Preterax and Diamicron MR Controlled Evaluation trial (Group et al., 2008), indicated that intensive treatment is paralleled by an improvement in renal function metrics; however, DN in a large population of diabetic patients continues to deteriorate after efficacious hypoglycemic therapy. Therefore, developing a new type of agent to improve both blood glucose and diabetic renal outcome seems increasingly urgent.

Proteinuria is usually accompanied by impairment of glomerular filtration. Podocytes, terminally differentiated visceral epithelial cells, are pivotal components in maintaining glomerular filtration barrier function and play a crucial role in the pathogenesis of proteinuria. Accumulating evidence indicates that detachment of podocytes from the glomerular basement membrane after apoptosis is the causative factor of proteinuria (Zhou et al., 2009). However, a recent study has demonstrated that significant proteinuria occurs ahead of podocyte detachment and apoptosis (Dai et al., 2006), arguing against the important role of podocyte apoptosis in the generation of proteinuria. A recent study proposed that podocyte epithelial-to-mesenchymal transition (EMT) was a potential pathway leading to proteinuria (Li et al., 2008). Podocyte EMT occurs when podocytes undergo phenotypic conversion (Li et al., 2008), characterized by the loss of the podocyte-specific markers nephrin and podocin and gain of transitional features, such as desmin, α-smooth muscle actin (α-SMA), fibronectin, and fibroblast-specific protein-1 (Fsp1) (Strutz et al., 1995; Li et al., 2008; Kang et al., 2010; Huang et al., 2014).

A host of studies have indicated that diabetic conditions result in podocyte injury, while this type of injury can be reversed through an anti-oxidative stress effect (Eid et al., 2016). Further studies have confirmed that oxidative stress is a crucial factor in the development of DN (Ratliff et al., 2016) and that the enhanced oxidative stress in diabetes can lead to podocyte dysfunction via the pro-oxidant enzyme NADPH oxidase and reactive oxygen species (ROS) production (Chen et al., 2013; Jha et al., 2016).

Dipeptidyl peptidase-4 (DPP-4) inhibitors, which can hinder degradation of bioactive incretins, such as glucagon-like peptide-1 (GLP-1), by inhibiting enhanced DPP-4 enzyme activity in diabetic conditions, have been developed as a new type of glycemic control agent in both clinical (Aschner et al., 2006; Sjostrand et al., 2014) and basic studies (Poucher et al., 2012). Interestingly, many experimental studies have shown that DPP4 inhibitors, such as linagliptin (Kanasaki et al., 2014) and alogliptin (Sakata et al., 2013), can improve albuminuria through diverse mechanisms, suggesting a favorable effect on kidney function. More importantly, the Saxagliptin Assessment of Vascular Outcomes Recorded in Patients with Diabetes Mellitus (SAVOR) has recently demonstrated the positive impact of saxagliptin on renal outcomes in patients with T2DM (Udell et al., 2015; Mosenzon et al., 2016). However, little is known about the mechanism by which saxagliptin alleviates albuminuria.

The DPP-4 enzyme is well known to be responsible for the cleavage of various substrates, such as GLP-1, brain natriuretic peptide (BNP), neuropeptide Y (NPY) and stromal cell-derived factor-1α (SDF-1α), which could affect additional consequences of DPP-4 inhibition beyond the hypoglycemic effect (Muskiet et al., 2014). SDF-1α, also known as CXCL12, is one of the most attractive DPP-4 physiological substrates because of its beneficial impact on pancreatic β cells (Yano et al., 2007), the heart (Kubota et al., 2016) and nerves (Sheu et al., 2012). Additionally, SDF-1α has recently been reported to have renal benefits according to a clinical study (Fujita et al., 2014b) and basic experiments (Takashima et al., 2016). However, thus far, there have been no studies on the effect of SDF-1α on podocyte EMT.

SDF-1α has been reported to induce activation of protein kinase A (PKA) (Yi et al., 2014), followed by inhibition of NADPH oxidase 2 (NOX2) (Fujita et al., 2014a), podocyte EMT through ROS production and ERK1/2 phosphorylation. In this context, we hypothesized that saxagliptin could reduce progressive albuminuria by preventing podocyte EMT in high-fat diet/streptozotocin (HFD)/STZ-induced type 2 diabetic rats via SDF-1α-mediated inhibition of renal NADPH oxidase.

## Materials and Methods

### Antibodies and Regents

The primary antibodies used in this study targeted SDF-1α (Novus Biologicals, Littleton, CO, United States), DPP-4 (Abcam, Cambridge, MA, United States), p-PKA (clone EP2606Y, Abcam, Cambridge, MA, United States), PKA (Cell Signaling, Danvers, MA, United States), NOX2 (clone EPR6991, Abcam, Cambridge, MA, United States), p-ERK1/2 (clone D13.14.4E, Cell Signaling, Danvers, MA, United States), ERK1/2 (Cell Signaling, Danvers, MA, United States), transforming growth factor-β1 (TGF-β1) (clone EPR18163, Abcam, Cambridge, MA, United States), podocin (Abcam, Cambridge, MA, United States), desmin (Abcam, Cambridge, MA, United States), nephrin (clone B-12, Santa Cruz Biotechnology Inc., Dallas, TX, United States), fibronectin (Proteintech, Rosemont, IL, United States), Fsp1 (clone EPR14639(2), Abcam, Cambridge, MA, United States), and α-SMA (clone 1A4, Abcam, Cambridge, MA, United States). ROS levels in renal tissue were tested with a dihydroethidium (DHE) assay kit (Beyotime, Jiangsu, China), and ROS levels in cells were examined with a DCFH-DA assay kit (Beyotime, Jiangsu, China). DPP-4 enzyme activity was tested using a DPP-4 assay kit (Enzo Life Sciences, Farmingdale, NY, United States).

### Animals

Eight-week-old male Sprague-Dawley rats (Beijing HFK Bioscience Co. Ltd, Beijing, China) were first randomly divided into 2 groups: a chow diet group (CD group, *n* = 15) group fed with regular rodent chow and HFD group (*n* = 30) allowed free access to the HFD for 10 weeks. Then, blood samples were collected to evaluate the induction of dyslipidemia and insulin resistance. The high-fat diet consisted of 78.7% standard diet, 10% sucrose, 10% animal fat, 1% cholesterol and 0.3% sodium cholate. After 10 weeks of chow and HFD diets, the homeostasis model assessment of insulin resistance (HOMA-IR) was examined to determine insulin resistance using an intraperitoneal glucose tolerance test (IPGTT). Subsequently, rats in the HFD group were injected with STZ (Sigma-Aldrich, St. Louis, MO, United States) in the tail (25 mg/kg body weight) to establish a T2DM model, while rats in the CD group were given an intravenous injection with citrate-phosphate buffer in the tail and served as the normal control group (NC group, *n* = 15). Establishment of the T2DM model was considered successful when the blood glucose level was more than 16.7 mmol/L. Diabetic rats were then randomly assigned to two groups: diabetic rats receiving vehicle only (DM group, *n* = 15) and diabetic rats orally administered saxagliptin daily (10 mg/kg body weight) (DM + Sax group, *n* = 15) by oral gavage for 12 weeks. The dose of saxagliptin was decided based on previous studies (Mason et al., 2011; Keller et al., 2015). All rats were sacrificed after 12 weeks of saxagliptin treatment. Saxagliptin was kindly provided by AstraZeneca. Body weight and blood glucose were examined each week. The study was approved by the ethical committee of Tianjin Medical University, and all procedures involving rats were conducted according to the Guide for the Care and Use of Laboratory Animals of the National Institutes of Health as well as the guidelines of the Animal Welfare Act.

### Cell Culture and Treatment

Conditionally immortalized mouse podocytes, MPC5, were kindly provided by Dr. Ming-zhen Li (Tianjin Metabolic Diseases Hospital) and were cultured under growth-permissive conditions at 33°C and 5% CO_2_. RPMI-1640 medium containing 10% FBS, 100 units/ml penicillin, 100 mg/ml streptomycin, and 20 units/ml mouse IFN-gamma (Peprotech) was utilized to maintain proliferative MPC-5 cells. Podocytes were exposed to 37°C without IFN-gamma for 10–14 days to induce differentiation.

Differentiated podocytes were cultured for 6–12 h in RPMI 1640 medium containing 5.6 mM D-glucose without FBS before exposure to various experimental conditions. The cells were then divided into four groups: (1) normal glucose (NG) group as controls incubated in RPMI 1640 containing 5.6 mM glucose, (2) mannitol (MA) group as an isosmotic control incubated in mannitol (24.4 mM) added to RPMI 1640 containing 5.6 mM glucose, (3) high glucose (HG) group incubated in RPMI 1640 containing 30 mM glucose, and (4) HG + Sax group incubated in HG medium (30 mM) and treated with 500 nM saxagliptin for 48 h. All of the glucose used in the present study was D-glucose from Sigma–Aldrich (St. Louis, MO, United States).

To determine the specific effect of the SDF-1α pathway on podocyte EMT, we further applied the SDF-1a receptor (CXCR4) antagonist AMD3100 (20 μg/ml) (Selleck Chemicals, Houston, TX, United States) for 1 h, followed by saxagliptin treatment or not under high-glucose (30 mM) conditions for 48 h. Cell lysates were collected for western blot analysis.

### Biochemical Analysis

We collected blood samples to assess serum lipid and renal and hepatic function as previously reported (Ma et al., 2015) before STZ injection and after saxagliptin treatment. At the end of the study, all rats were placed in individual metabolic cages to collect 24-h urine samples for measurement of urinary microalbumin (UMA) excretion and urinary chemistries. Urinary UMA was examined with an automatic biochemistry analyzer (Roche). The urinary 8-hydroxy-2′-deoxyguanosine (8-OHdG) and mindin levels were measured with commercially available enzyme-linked immunosorbent assay (ELISA) kits (Biotopped, Beijing, China). Rats were sacrificed under anesthesia by intraperitoneal injection of chloral hydrate. Blood samples were obtained from the retroorbital venous plexus at sacrifice and were centrifuged at 3000 rpm/min for 10 min. Total triglyceride (TG), total cholesterol (TC), blood urea nitrogen (BUN), serum creatinine (Scr), aspartate transaminase (AST), and alanine transaminase (ALT) in serum were tested with an automatic biochemistry analyzer (Roche). Then, the left kidneys were immediately removed, weighed, and frozen in liquid nitrogen before being stored at -80°C or fixed in 4% paraformaldehyde.

### Intraperitoneal Glucose Tolerance Test

Intraperitoneal glucose tolerance tests (IPGTT) were performed to examine the existence of insulin resistance after 10 weeks of a HFD. Rats in both the CD group and the HFD group were fasted overnight, and then, glucose was injected intraperitoneally (2 g/kg body weight). Blood samples were drawn at the indicated time points. The glucose and insulin levels were measured with a glucometer and an ELISA kit respectively. Subsequently, HOMA-IR was assessed to evaluate insulin resistance using the following formula: HOMA-IR = fasting glucose (mmol/l) × fasting insulin (μU/ml)/22.5 (Konrad et al., 2007).

### ROS Detection

To detect ROS production in renal glomeruli, DHE staining was performed after the rat kidneys were immediately frozen in O.C.T. compound (Tissue-Tech II; Sakura Fine Chemical, Tokyo, Japan) and sectioned to a 4 μm thickness on a cryostat. Fluorescence images were obtained using a fluorescence microscope. Intracellular ROS production was detected with the DCFH-DA assay. The cells were cultured in 24-well plates before a series of treatments. Cells were then treated with 10 μmol/L DCFH-DA in serum-free RPMI 1640 medium for 30 min at 37°C. After being washed three times with serum-free RPMI 1640 medium, the cells were observed under a fluorescence microscope. Positive staining was determined using Image-Pro plus 6.0 image analysis software.

### Western Blot Analysis

Protein lysate from either kidneys or cells was denatured by boiling in SDS sample buffer at 100°C for 10 min. After transferring protein from gels onto PVDF membranes, the membranes were immersed in TBS-T containing 5% non-fat milk for 2 h at room temperature before incubation with primary antibodies at 4°C overnight and subsequent incubation with secondary antibodies at room temperature for 2 h. The primary antibodies targeted SDF-1α (1:2000), DPP-4 (1:2000), p-PKA (1:2000), PKA (1:2000), NOX2 (1:3000), p-ERK1/2 (1:2000), ERK1/2 (1:2000), TGF-β1 (1:1000), α-SMA (1:300), podocin (1:1000), desmin (1:1000), and nephrin (1:300). The target bands on membranes were visualized with ECL. Band intensity was quantified using ImageJ software and normalized to the respective control.

### Hematoxylin-Eosin Staining and Periodic Acid–Schiff Staining of Renal Tissues

After the renal tissue was fixed in 4% paraformaldehyde, the kidneys were embedded in paraffin and cut into sections (4 μm). After deparaffinization, hematoxylin-eosin (HE) staining and periodic acid-Schiff (PAS) staining were performed. Changes in 10 randomly selected glomeruli were observed with a light microscope (×400). The mesangial area was evaluated with Image-Pro Plus 6.0 image analysis software.

### Transmission Electron Microscopy Detection

From the renal tissues, 1 mm^3^ of tissue was obtained and immediately prefixed in 2.5% glutaraldehyde, followed by 1% osmium tetroxide. Subsequently, tissues were dehydrated in a series of alcohols and finally embedded. Ultrastructural changes of podocytes were observed under a transmission electron microscope.

### Immunohistochemistry

After deparaffinization, heat-induced antigen retrieval was conducted at 95°C by microwave in 10 mmol/L sodium citrate buffer (pH6.0) for 5 min. After that, the sections were blocked with 3% H_2_O_2_ for 15 min and incubated with primary antibodies at 4°C overnight. The primary antibodies were diluted with phosphate-buffered saline (PBS) and anti-fibronectin (1:200), anti-Fsp1 (1:200), anti-SDF-1α (1:500), and anti-NOX2 (1:200). After that, the sections were incubated with HRP-conjugated secondary antibodies for 30 min at 37°C. After staining with a diaminobenzidine (DAB) kit, sections were counterstained with hematoxylin. Stained images (10 glomeruli per kidney) were captured with a light microscope in a blinded manner at a magnification × 400, and all the images were quantified with Image Pro Plus 6.0 software.

### Immunofluorescence

For immunofluorescence staining of desmin, nephrin and podocin, frozen sections of the kidney were used. In brief, slides were first blocked with BSA and then incubated with primary antibodies. For immunofluorescence staining of DPP-4, paraffin-embedded kidney sections were used, and the protocol was similar to the procedure described for immunohistochemistry with a few modifications. The primary antibodies used targeted desmin (1:100), nephrin (1:50), podocin (1:100), and DPP-4 (1:100). After incubation with secondary antibodies at 37°C, fluorescence images were captured with a fluorescence microscope (×400). All data were calculated from images of 10 randomly selected glomeruli per kidney using Image Pro Plus 6.0 software.

### Statistical Analysis

The data are expressed as the mean ± standard deviation (SD). Statistical analysis between two groups was performed using two-tailed, unpaired Student’s *t*-test. One-way analysis of variance (ANOVA) was used for statistical comparison among more than three groups, and Tukey’s *post hoc* test was performed between any two groups. *P* < 0.05 was considered statistically significant.

## Results

### Saxagliptin Improved Excretion of Urinary Microalbumin and a Podocyte Injury Marker

**FIGURE 1:**
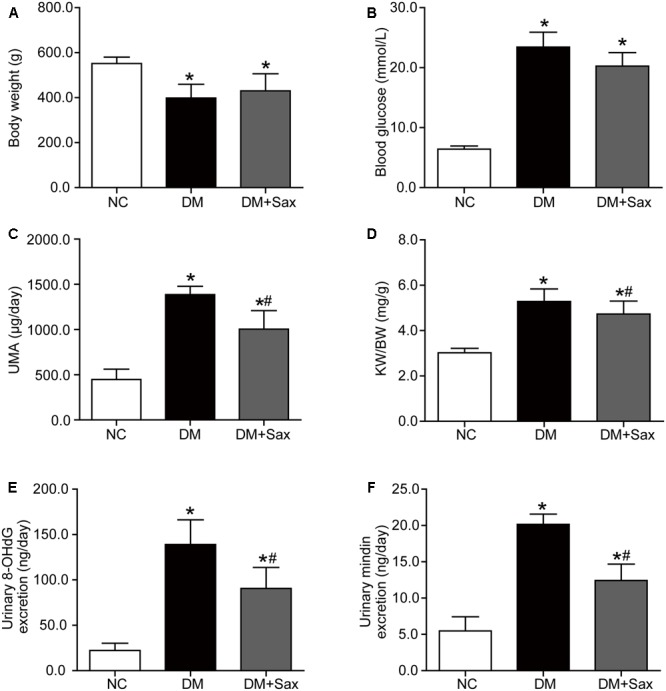
Effects of saxagliptin treatment on characteristics of HFD/STZ-induced diabetic rats. **(A–D)** Body weight, blood glucose, UMA and KW/BW in the NC group, the DM group and the DM + Sax group after 12-week saxagliptin treatment. **(E,F)** Urinary oxidative stress marker 8-OHdG levels and urinary podocyte injury marker mindin levels. One-way ANOVA followed by Tukey’s test was used to compare the differences between any two of the three groups. The data are presented as the mean ± SD (*n* = 12 for each group). ^∗^*P* < 0.05 vs. NC group; ^#^*P* < 0.05 vs. DM group. NC, non-diabetic group as normal control; DM, non-treated diabetic group; DM + Sax, diabetic rats with saxagliptin administration; UMA, urinary microalbumin; KW/BW, kidney weight-to-body weight ratio; 8-OHdG, 8-hydroxy-2′-deoxyguanosine.

After rats were administered a HFD for 10 weeks, there was obvious insulin resistance in the HFD group as illustrated by IPGTT and HOMA-IR (Supplementary Figures 1C,D). The body weight and blood lipids in the HFD group were significantly higher compared with those in the CD group (Supplementary Figures 1A,E), whereas the blood glucose, hepatic enzyme and renal function were not changed between the two groups (Supplementary Figures 1B,F,G). After the HFD/STZ–induced T2DM model was established followed by 12 weeks of saxagliptin treatment, we observed that saxagliptin did not significantly affect the lipid profiles, hepatic function or serum creatinine levels in diabetic rats (Supplementary Figures 2A–C). Moreover, saxagliptin treatment did not cause any alterations in body weight or blood glucose, though it tended to decrease blood glucose, in the DM + Sax group compared with the DM group (**Figures 1A,B**). The UMA levels (**Figure 1C**), kidney weight-to-body weight ratio (KW/BW) (**Figure 1D**) were notably higher in the DM group compared to the NC group, while they were obviously suppressed after saxagliptin treatment. To explore the mechanism underlying these beneficial effects of saxagliptin, we tested the excretion levels of the systemic oxidative stress marker 8-OHdG and podocyte injury marker mindin (Murakoshi et al., 2011). The results showed that the diabetes-induced increases of both the 8-OHdG and mindin levels were dramatically prevented to some degree after saxagliptin administration, which showed the possible mechanism of saxagliptin in renal protection (**Figures 1E,F**).

### Saxagliptin Improved Podocyte Structure in Renal Tissue

**FIGURE 2:**
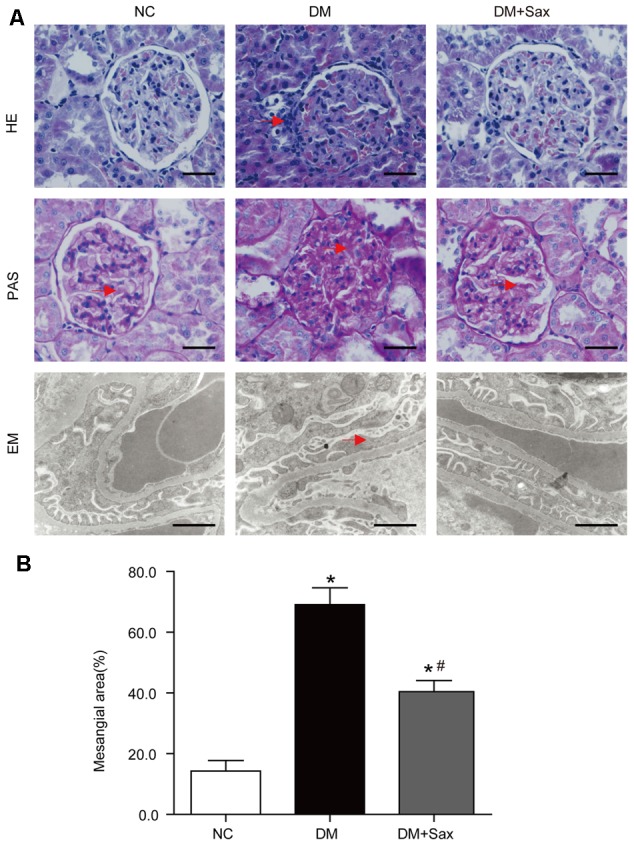
Effects of saxagliptin treatment on podocyte pathological changes. **(A)** Representative photomicrographs of renal histology and ultrastructure of podocytes, **(B)** Mesangial area expansion analysis. Renal histology changes were identified with HE staining (original magnification × 400, bars = 50 μm), and extracellular matrix accumulation in glomeruli was determined with PAS staining (original magnification × 400 bars = 50 μm). The podocyte structure was observed with transmission EM (bars = 1 μm). Red arrows indicate the inflammatory cell infiltration in HE-stained tissue, deposited mesangial matrix in the PAS-stained tissue, and foot process effacement in EM images. One-way ANOVA followed by Tukey’s test was used to compare the differences between any two of the three groups. The data are presented as the mean ± SD (*n* = 5 for each group). ^∗^*P* < 0.05 vs. NC group; ^#^*P* < 0.05 vs. DM group. NC, non-diabetic group as normal control; DM, non-treated diabetic group; DM + Sax, diabetic rats with saxagliptin administration; HE, hematoxylin-eosin staining; PAS, periodic acid–Schiff staining; EM, electron microscopy.

Hematoxylin and eosin (HE), periodic acid–Schiff (PAS) staining and transmission electron microscopy were performed to examine pathologic changes in the kidney. The light microscopy images revealed that the deposited mesangial matrix, mesangial expansion, and fractional mesangial area were significantly higher in the DM group than in the NC group and were reversed by saxagliptin treatment (**Figures 2A,B**). Transmission electron microscopy visually revealed the ultrastructure of podocytes. It showed that the foot processes (FPs) in the NC group were well arranged and distributed. However, a portion of the FPs in the DM group exhibited obvious effacement, which was notably improved by saxagliptin treatment (**Figure 2A**).

### Saxagliptin Inhibited Diabetes-Induced Podocyte EMT in Renal Tissue

**FIGURE 3:**
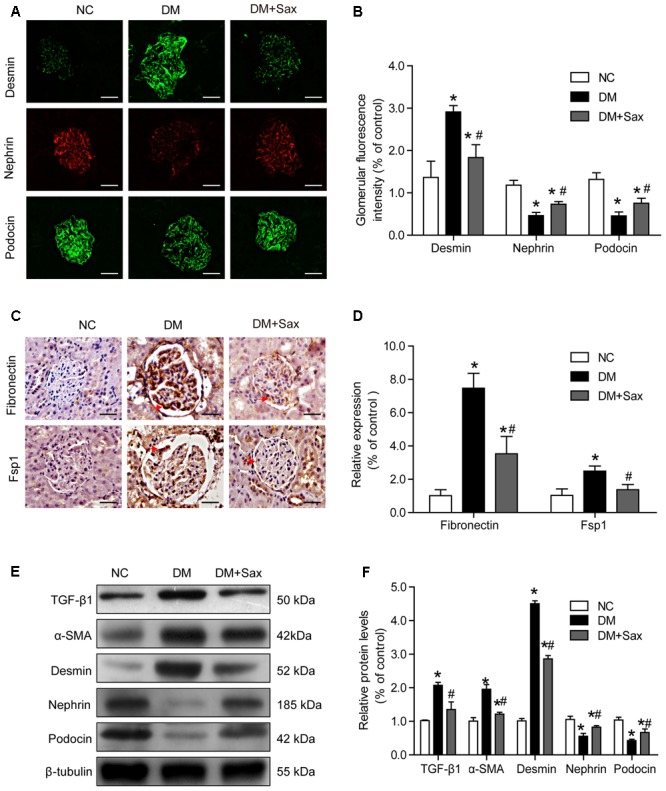
Effects of saxagliptin treatment on podocyte EMT in HFD/STZ-induced diabetic rats. **(A,B)** Immunofluorescence analysis of desmin, nephrin, and podocin (original magnification × 400, bars = 50 μm), as well as their quantification. **(C,D)** Immunochemistry analysis of fibronectin and FSP1 (original magnification × 400, bars = 50 μm), as well as their quantification. **(E,F)** Western blot analysis of TGF-β1, α-SMA, desmin, nephrin, and podocin and densitometric analysis of the expression of each protein after 12 weeks of saxagliptin treatment. One-way ANOVA followed by Tukey’s test was used to compare the differences between any two of the three groups. The data are presented as the mean ± SD (*n* = 5 for each group). ^∗^*P* < 0.05 vs. NC group; ^#^*P* < 0.05 vs. DM group. NC, non-diabetic group as normal control; DM, non-treated diabetic group; DM + Sax, diabetic rats with saxagliptin administration.

To verify whether saxagliptin protected podocytes, we examined the expression of the podocyte injury indicator desmin and podocyte slit diaphragm components, including nephrin and podocin, using immunofluorescence staining. We found that desmin expression was significantly increased in the DM group compared with the NC group, while it was notably decreased in the DM + Sax group (**Figures 3A,B**). In addition, expression of nephrin and podocin was dramatically elevated in the DM + Sax group compared with the DM group (**Figures 3A,B**). Through immunochemistry, we observed that saxagliptin significantly decreased expression of the EMT markers fibronectin and Fsp1 in the glomerulus of diabetic rats (**Figures 3C,D**). Moreover, western blot analysis revealed that the potent EMT inducer TGF-β1 and EMT marker α-SMA were expressed at significantly higher levels in the DM group than in the NC group, while obviously lower expression was observed in the DM+Sax group compared with the DM group (**Figures 3E,F**). A trend similar to that observed with immunofluorescence staining was observed for protein expression of desmin, nephrin and podocin (**Figures 3E,F**).

### Saxagliptin Inhibited DPP-4 Activity/Expression and SDF-1α Cleavage in Renal Tissue

**FIGURE 4:**
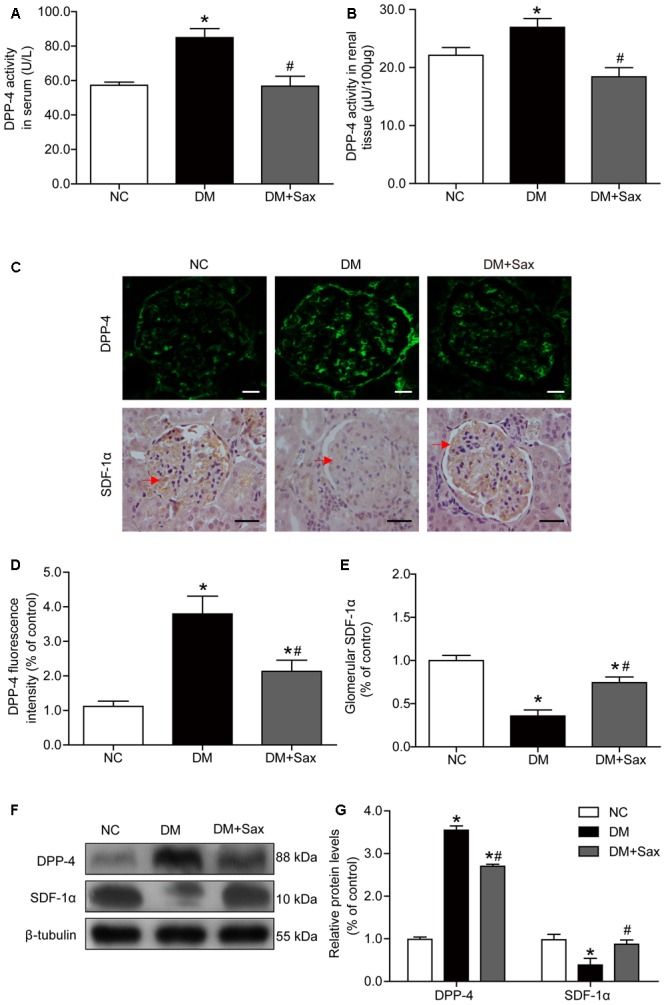
Effects of saxagliptin treatment on DPP-4 activity/expression and SDF-1α levels in HFD/STZ-induced diabetic rats. **(A,B)** DPP-4 enzyme activity respectively in serum and renal tissue. **(C)** Immunofluorescence and immunohistochemistry analysis for DPP-4 and SDF-1α expression respectively (original magnification × 400, bars = 20 μm for DPP-4 images or bars = 50 μm for SDF-1α images). **(D,E)** Quantification of DPP-4 and SDF-1α expression by immunofluorescence/immunohistochemistry. **(F)** Western blot analysis for protein expression of DPP-4 and SDF-1α. **(G)** Densitometric analysis of indicated protein expression relative to β-tubulin levels. One-way ANOVA followed by Tukey’s test was used to compare the differences between any two of the three groups. The data are presented as the mean ± SD (*n* = 5 for each group). ^∗^*P* < 0.05 vs. NC group; ^#^*P* < 0.05 vs. DM group. NC, non-diabetic group as normal control; DM, non-treated diabetic group; DM + Sax, diabetic rats with saxagliptin administration.

As we speculated, saxagliptin might protect podocytes by inhibiting DPP-4 enzyme activity and preventing SDF-1α cleavage; therefore, we determined the DPP-4 enzyme activity in both serum and renal tissue and found that saxagliptin obviously inhibited the diabetes-enhanced DPP-4 enzyme activity in diabetic rats (**Figures 4A,B**). As illustrated in **Figures 4C–E**, DPP-4 expression was mostly localized to the glomerulus based on immunological staining, and rats in the DM group exhibited significantly increased DPP-4 expression compared with rats in the NC group, while the DM + Sax group exhibited a markedly lower expression compared with the DM group. As we hypothesized, immunohistochemistry analysis showed that a notably higher level of the important substrate of DPP-4, SDF-1α, was found in the renal tissue in the DM + Sax group compared with that in the DM group. Western blotting revealed a trend in both DPP-4 and SDF-1α protein expression similar to that observed with immunological staining (**Figures 4F,G**).

### Saxagliptin Attenuated Oxidative Stress via the PKA/NOX2/ERK Pathway in Renal Tissue

**FIGURE 5:**
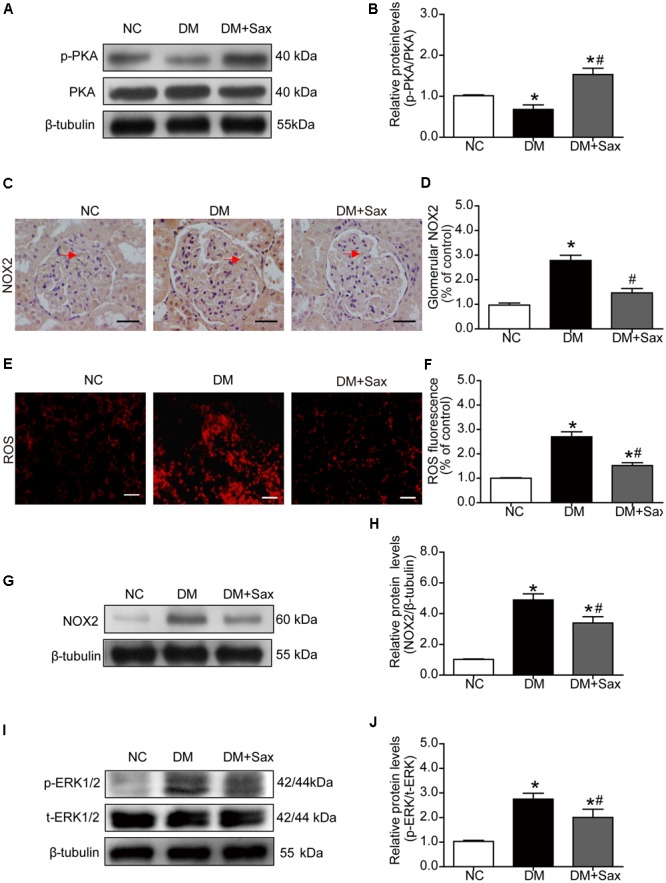
Effects of saxagliptin treatment on PKA phosphorylation and oxidative stress in HFD/STZ-induced diabetic rats. **(A)** Expression of PKA phosphorylation was assessed by Western blot analysis. **(B)** Quantification of PKA phosphorylation. **(C–F)** Immunohistochemistry and dihydroethidium staining for NOX2 expression (original magnification × 400, bars = 50 μm) and ROS production (original magnification × 200, bars = 50 μm) as well as their quantification of expression. **(G)** Representative Western blot for NOX2 expression in rats. **(H)** Quantification of NOX2 revealed by Western blot. **(I,J)** Western blot result for phosphorylation of ERK1/2 as well as its quantification. One-way ANOVA followed by Tukey’s test was used to compare the differences between any two of the three groups. The data are presented as the mean ± SD (*n* = 5 for each group). ^∗^*P* < 0.05 vs. NC group; ^#^*P* < 0.05 vs. DM group. NC, non-diabetic group as normal control; DM, non-treated diabetic group; DM + Sax, diabetic rats with saxagliptin administration.

Because a major downstream pathway of SDF-1α is activation of PKA, we examined phosphorylation of PKA. We noted that the phosphorylated PKA levels in the DM group were significantly decreased compared with those in the NC group and notably increased after saxagliptin treatment (**Figures 5A,B**). NADPH oxidases have been reported to be inhibited by PKA activation (Fujita et al., 2014a), and NADPH oxidase is major source of ROS in the kidney (Li and Shah, 2003). The immunohistochemistry and DHE staining results were consistent with the notion that NOX2 and ROS expression was markedly increased in both tubules and glomeruli in the DM group compared with the NC group, while saxagliptin markedly reduced these levels nearly back to normal levels (**Figures 5C–F**). NOX2 protein expression was further confirmed by western blotting (**Figures 5G,H**). As a regulator in the TGF-β1 signaling pathway (Rhyu et al., 2005; He et al., 2015), we examined the phosphorylated ERK1/2 protein levels and found that they were significantly upregulated in the DM group compared with the NC group and obviously down-regulated following 12 weeks of saxagliptin treatment (**Figures 5I,J**).

### Saxagliptin Inhibited DPP-4 Activity/Expression and SDF-1α Protein Cleavage in Cultured Podocytes

**FIGURE 6:**
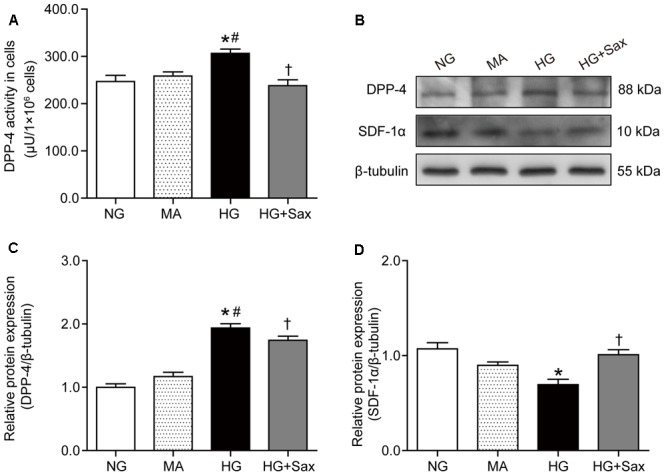
Effects of saxagliptin treatment on DPP-4 inhibition and SDF-1α protein cleavage in cultured podocytes. **(A)** DPP-4 enzyme activity measurements in podocytes. **(B)** Protein expression of DPP-4 and SDF-1α in respectively NG as controls, MA group as an isoosmotic control, HG group, and HG group with saxagliptin treatment. **(C,D)** Quantification of DPP-4 and SDF-1α expression. One-way ANOVA followed by Tukey’s test was used to compare the differences between any two of the three groups. The data are presented as the mean ± SD (*n* = 3–4 for each group, 3 replicates). ^∗^*P* < 0.05 vs. NG group; ^#^*P* < 0.05 vs. MA group; ^†^*P* < 0.05 vs. HG group. NG, normal glucose; MA, mannitol; HG: high glucose, Sax, saxagliptin.

To further confirm the preventive effect of saxagliptin against podocyte EMT, studies were carried out in podocytes exposed to various conditions. We observed that saxagliptin significantly suppressed high-glucose-induced increased DPP-4 enzyme activity/protein expression compared with NG treatment, whereas mannitol had no effect (**Figures 6A–C**). We also detected the protein expression of SDF-1α and found that it was markedly decreased in high-glucose conditions, possibly by DPP-4 enzyme cleavage, while significantly high levels were exhibited after saxagliptin treatment (**Figures 6B,D**).

### Saxagliptin Inhibited High-Glucose-Induced Podocyte EMT via the PKA/NOX2/ERK Pathway in Cultured Podocytes

**FIGURE 7:**
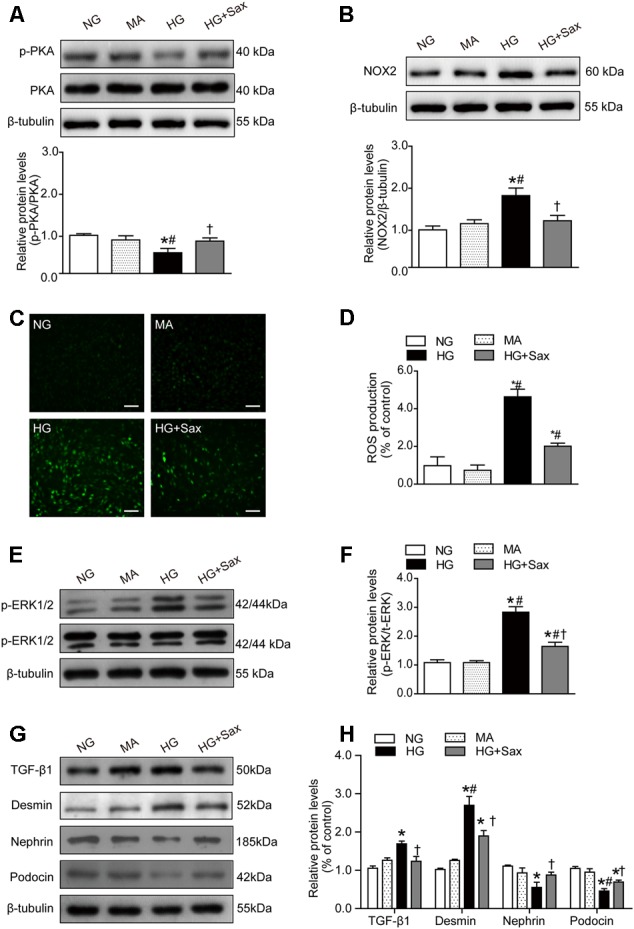
Effects of saxagliptin treatment on oxidative stress and podocyte EMT in cultured podocytes. **(A)** Protein expression of PKA phosphorylation as well as its quantification respectively in NG as controls, MA group as an isoosmotic control, HG group, and HG group with saxagliptin treatment. **(B)** NOX2 expression and its quantification in various conditions. **(C,D)** The amount of ROS production in podocytes detected by DCFH-DA assay (original magnification × 200, bars = 100 μm). **(E,F)** The protein expression of ERK1/2 phosphorylation assessed by Western blot analysis from cell lysates in different conditions and its quantification. **(G,H)** Western blot analysis for TGF-β1, desmin, nephrin, and podocin in various conditions and their quantifications. One-way ANOVA followed by Tukey’s test was used to compare the differences between any two of the three groups. The data are presented as the mean ± SD (*n* = 3–4 for each group, 3 replicates). ^∗^*P* < 0.05 vs. NG group; ^#^*P* < 0.05 vs. MA group; ^†^*P* < 0.05 vs. HG group. NG, normal glucose; MA, mannitol; HG: high glucose, Sax, saxagliptin.

To explore the effect of saxagliptin on the downstream SDF-1α pathway, we examined the level of PKA phosphorylation as well as NOX2 and ROS production. The results showed that the PKA phosphorylation levels were clearly decreased in high-glucose-treated podocytes and significantly increased after saxagliptin treatment (**Figure 7A**). It was also observed that saxagliptin treatment notably attenuated HG-enhanced NOX2 protein expression and ROS levels, as it did in the diabetic rats (**Figures 7B–D**). Subsequently, we investigated the ERK1/2 phosphorylation levels and EMT and observed that high-glucose conditions significantly increased the levels of phosphorylated ERK1/2, the EMT inducer TGF-β1, and the podocyte injury indicator desmin and dramatically reduced protein expression of nephrin and podocin, while these changes were remarkably restored by saxagliptin (**Figures 7E–H**).

### Saxagliptin Inhibited Podocyte EMT through SDF-1α via the PKA/NOX2/ERK Pathway in Cultured Podocytes

**FIGURE 8:**
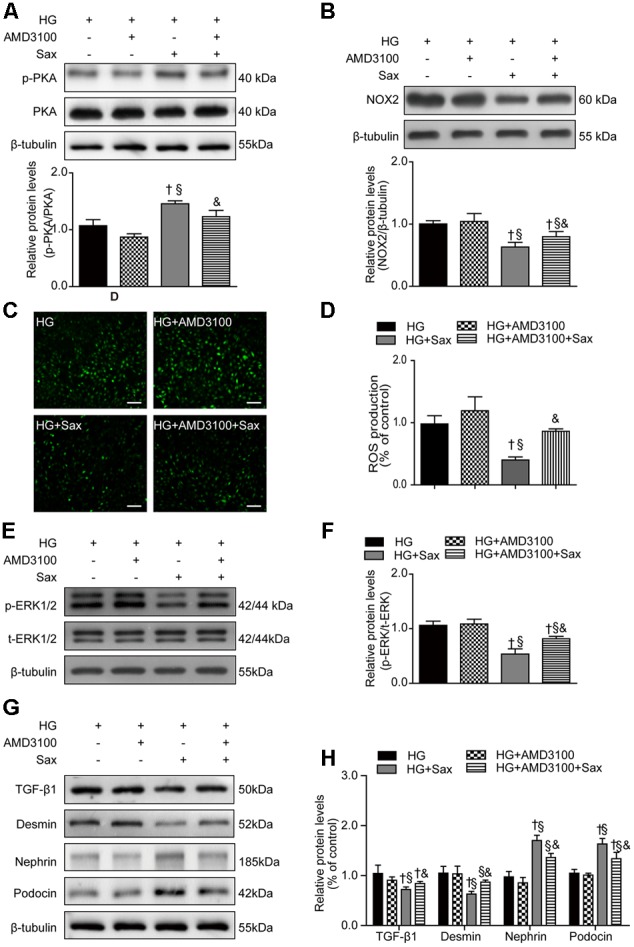
Effects of SDF-1α receptor blockade via AMD3100 followed by saxagliptin treatment on oxidative stress and podocyte EMT in cultured podocytes. **(A)** Western blot analysis of the PKA phosphorylation levels and their quantification after treatment with HG, HG plus AMD3100, HG with saxagliptin, and pretreatment with AMD3100 for 1–2 h followed by saxagliptin under HG conditions. **(B)** Western blot analysis of NOX2 and its quantification in various conditions. **(C,D)** The amount of ROS production in podocytes detected with a DCFH-DA assay (original magnification × 200, bars = 100 μm). **(E,F)** Protein expression of phosphorylated ERK1/2 assessed by Western blot analysis of cell lysates under different conditions and its quantification. **(G,H)** Western blot analysis of TGF-β1, desmin, nephrin, and podocin under various conditions and their quantification. One-way ANOVA followed by Tukey’s test was used to compare the difference between any two of the four groups. The data are presented as the mean ± SD (*n* = 3–4 for each group, 3 replicates). ^†^*P* < 0.05 vs. HG group; ^§^ *P* < 0.05 vs. HG+AMD3100 group; ^&^*P* < 0.05 vs. HG + Sax group. HG, high glucose; Sax, saxagliptin.

To confirm the specific function of SDF-1α, AMD3100 was applied to cultured podocytes prior to saxagliptin treatment. We noted no significant difference in the levels of PKA phosphorylation between the HG and HG + AMD3100 groups, while the PKA phosphorylation levels were obviously increased after saxagliptin treatment and were markedly abrogated in the HG + AMD3100 + Sax group (**Figure 8A**). Furthermore, NOX2 expression and ROS production were unchanged in the HG + AMD3100 group compared with the HG group, while they were evidently reduced in the HG + Sax group and significantly enhanced when AMD3100 was used to block the SDF-1α signaling pathway (**Figures 8B–D**). Moreover, in the HG + Sax group, the phosphorylation levels of ERK1/2 and protein levels of TGF-β1 and desmin were evidently decreased compared with those of the HG group and HG + AMD3100 group, while the expression of nephrin and podocin was dramatically increased, and AMD3100 remarkably blocked this effect (**Figures 8E–H**), indicating that AMD3100 significantly blocked the protective effect of saxagliptin in podocyte EMT through the PKA/NOX2/ERK signaling pathway and that the SDF-1α pathway was involved in high-glucose-induced EMT in podocytes.

## Discussion

The present study is the first to demonstrate that saxagliptin treatment can prevent podocyte EMT in HFD/STZ-induced T2DM rats, as evidenced by decreased excretion of microalbumin and the urinary podocyte injury marker mindin (Murakoshi et al., 2011) beyond its anti-hyperglycemic effect (Uchii et al., 2016) as well as significantly suppressed renal oxidative stress. Clearly, we showed a remarkable improvement in podocyte histology using transmission electron microscopy. We also showed that DPP-4 activity/protein was significantly increased in the renal tissue of T2DM rats and high glucose cultured podocytes, which was considered a marker of renal injury in several recently reported studies (Sun et al., 2012; Matsui et al., 2015; Zheng et al., 2016). Notably, following saxagliptin treatment *in vivo* and *in vitro*, DPP-4 activity/protein expression was significantly decreased, accompanied by the obvious restoration of the SDF-1α levels and PKA signaling pathway activation, which could account for the subsequent repression of renal oxidative stress and TGF-β1 expression. In addition, saxagliptin-prevented podocyte EMT was notably blunted when the SDF-1α pathway was blocked with AMD3100. Here, our data demonstrated a novel renoprotection function of saxagliptin through the SDF-1α activation-related PKA/NOX2 pathway.

It is well known that podocytes play an important part in proteinuria, and EMT (Li et al., 2008) emphasizes the phenotypic alterations of podocytes rather than their loss, which is the primary cause of the podocyte dysfunction that leads to proteinuria in many common glomerular diseases. TGF-β1 is a profibrotic cytokine found in chronic renal disease and is reported to induce EMT in cells, such as podocytes (Loeffler and Wolf, 2014). α-SMA is the marker protein of myofibroblasts. By producing the interstitial matrix component fibronectin, podocytes have adapted a mesenchymal phenotype. Fsp1 is a fibroblast-specific protein (Strutz et al., 1995). Desmin is a type of intermediate filament protein and has long been considered a podocyte injury indicator (Zou et al., 2006; Kakimoto et al., 2014). In addition, induction of desmin is regarded as a common feature of mesenchymal cell activation in different organs (Huang et al., 2014; Velickov et al., 2016). Given these lines of evidence, it is conceivable that these proteins are used as podocyte EMT markers in studies (Kang et al., 2010). It is well-established that nephrin and podocin are important components of the slit diaphragm in podocytes, and their loss will certainly destroy the integrity of the slit diaphragm, thereby resulting in proteinuria (Brinkkoetter et al., 2013). In the present study, we first observed that saxagliptin remarkably diminished high-glucose-induced high expression of TGF-β1, α-SMA, desmin, fibronectin, and Fsp1 and notably improved nephrin/podocin expression *in vivo* and *in vitro*, indicating that saxagliptin may prevent podocyte EMT. Furthermore, AMD3100 is a chemokine receptor antagonist against the binding of CXCR4 to its ligand SDF-1α, and AMD3100 in the podocyte experiment was observed to have an obvious adverse impact on the expression of these proteins compared with saxagliptin treatment, further verifying that the protective role of SDF-1α on podocyte EMT may be a mechanism through which saxagliptin prevents albuminuria.

DPP-4 is well known to have a number of substrates, including GLP-1, that may account for its favorable effect (Drucker, 2007). In this regard, recent data have suggested that SDF-1α was upregulated by DPP-4 inhibition and has protective roles in progressive DN (Takashima et al., 2016). Previous studies have reported that SDF-1α levels were significantly reduced in both STZ-induced diabetic mice (Gallagher et al., 2007; Li et al., 2012) and diabetic patients (Angela Castela et al., 2017) compared to the normal groups, while it was markedly increased in the DPP-4 inhibitor treatment group (Fadini et al., 2010; Fujita et al., 2014b; Lovshin et al., 2017). In parallel with SDF-1α upregulation, DPP-4 inhibitor treatment clearly attenuated the progression of albuminuria not only in animal experiments (Nistala et al., 2014) but also in clinical studies (Fujita et al., 2014b). This may be due to rapid cleavage of SDF-1α induced by enhanced DPP-4 enzyme activity in high-glucose conditions and the suppressive effect that DPP-4 inhibitors have on cleavage through inhibiting DPP-4 activity in chronic diabetic conditions. Furthermore, an experimental mouse study has revealed that DPP-4 inhibition upregulated SDF-1α expression in the diabetic kidneys in a GLP-1R-independent manner (Takashima et al., 2016). The above evidence indicated the protective role of SDF-1α in renal protection. In line with previous studies, our study revealed that saxagliptin notably diminished the high-glucose enhanced activity/protein expression of DPP-4 in diabetic rats/cultured podocytes and subsequently dramatically restored the SDF-1α levels. Our observation suggested that saxagliptin inhibited SDF-1α cleavage through DPP4 inhibition, and SDF-1α may serve as a pivotal mediator in renal protection.

A previous study has shown that SDF-1α induced activation of PKA through its G-protein coupled receptor (GPCR), and it is well established that PKA is the main downstream effector of GPCR signaling pathways (Zha et al., 2015). Moreover, experiments have suggested that NOX2 was reduced by activation of the PKA signaling pathway (Fujita et al., 2014a). NADPH oxidase is a major source of ROS in certain renal cells (Li and Shah, 2003), and ROS are reported to mediate TGF-β1-induced EMT through activation of ERK1/2 in tubular epithelial cells (Rhyu et al., 2005; He et al., 2015). Therefore, a PKA activation-induced decrease in NOX2 may lead to attenuation of ROS production, followed by decreased ERK1/2 phosphorylation and podocyte EMT. In our animal and podocyte experiments, we found that in parallel with the high levels of renal SDF-1α after saxagliptin treatment, phosphorylated PKA levels were remarkably elevated, while the levels of NOX2, ROS, ERK1/2 phosphorylation and EMT were clearly reduced. This observation confirmed the SDF-1α/PKA signaling pathway in renal tissue and podocytes. Therefore, the high levels of SDF-1α induced by DPP-4 inhibition may lead to podocyte EMT attenuation through PKA pathway activation and a subsequent reduction in renal oxidative stress. Additionally, the increased expression of these oxidative stress markers induced by AMD3100 further confirmed the specific effect of SDF-1α.

## Conclusion

**FIGURE 9:**
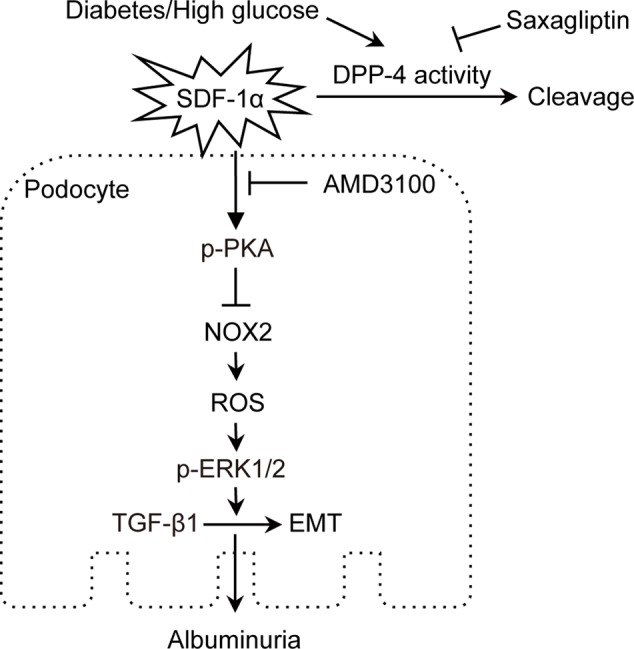
Proposed mechanism of podocyte EMT inhibition by saxagliptin. Saxagliptin treatment prevents SDF-1α cleavage by inhibiting enhanced DPP-4 enzyme activity and contributes to a reduction in oxidative stress markers, including NOX2, ROS and phosphorylated ERK1/2, by activating PKA phosphorylation, which hinders podocyte EMT and attenuates albuminuria. SDF-1α receptor blockade with AMD3100 blocks the function of saxagliptin in podocyte EMT, suggesting an SDF-1α role in preventing albuminuria through inhibition of podocyte EMT *in vivo* and *in vitro*.

As summarized in **Figure 9**, our study demonstrated for the first time that saxagliptin treatment hindered podocyte EMT by preventing SDF-1α cleavage and subsequent inhibition of oxidative stress. Because proteinuria occurs in the early stage of DN and podocytes may undergo EMT in the early DN state, administering effective treatment as early as possible may help prevent the development of DN. This study will broaden our awareness of the positive effects of saxagliptin on progressive DN. Further studies are needed to explore whether other mechanisms also participate in the renal protection effect of saxagliptin in DN.

## Author Contributions

Y-pC contributed to acquisition and interpretation of data, and to writing the manuscript; BS and ZH contributed to expert study design and data analysis; FH, S-lH, Xy-L, MX, YY, and LC contributed to experimental assistance; C-jL contributed to the study design and proofreading of the manuscript; L-mC contributed to the study design, acquisition of data, revision of the manuscript, and approval of the version to be submitted.

## Conflict of Interest Statement

The authors declare that the research was conducted in the absence of any commercial or financial relationships that could be construed as a potential conflict of interest.
