# Supplementary material for: Saxagliptin Attenuates Albuminuria by Inhibiting Podocyte Epithelial- to-Mesenchymal Transition via SDF-1α in Diabetic Nephropathy
Source: Front Pharmacol. 2017 Nov 1;8:780. doi: 10.3389/fphar.2017.00780 (PMC5672017; doi:10.3389/fphar.2017.00780)
Supplement: Supplementary file 1 [file Presentation_1.ppt]

## Slide 1
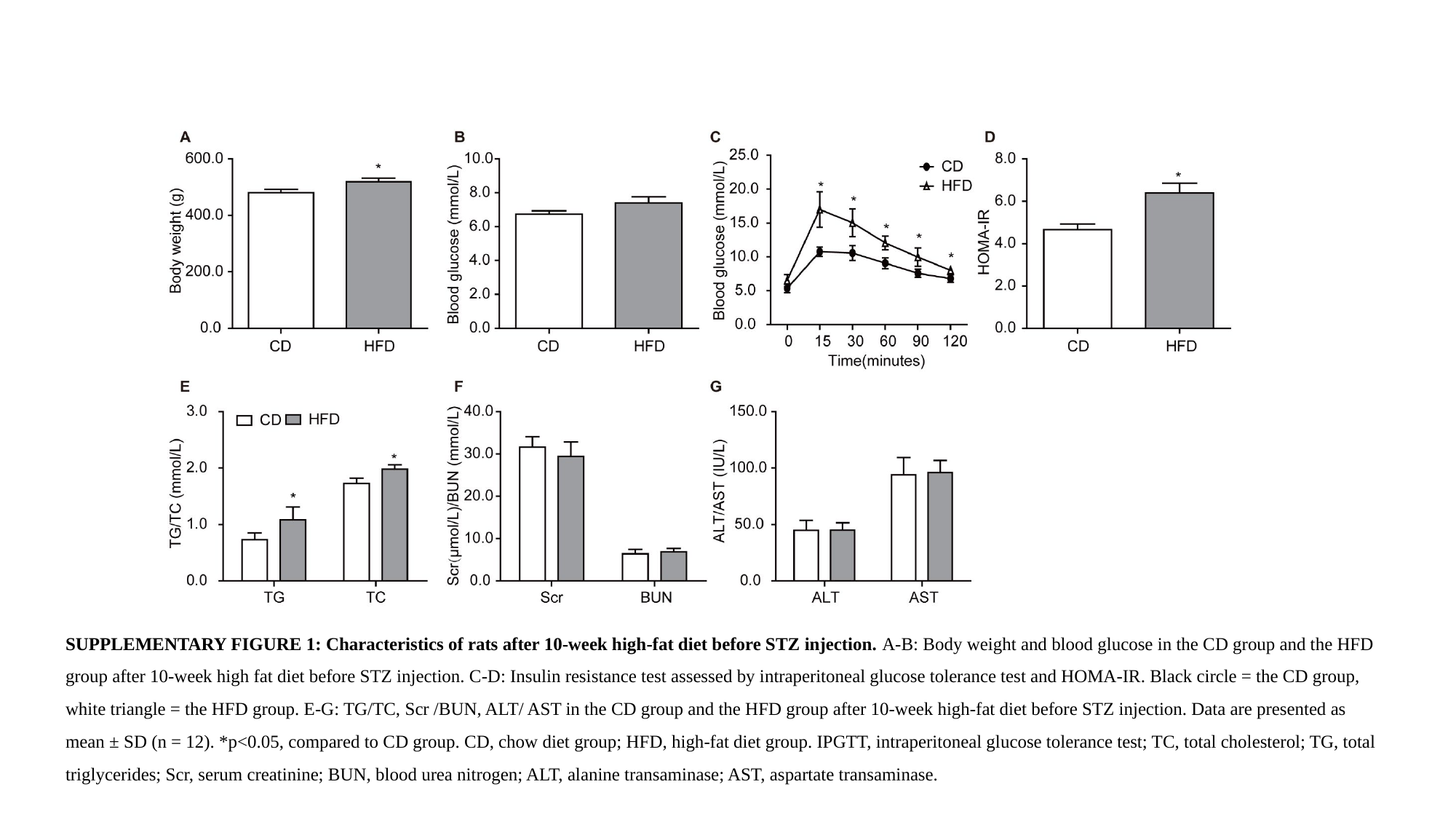

SUPPLEMENTARY FIGURE 1: Characteristics of rats after 10-week high-fat diet before STZ injection. A-B: Body weight and blood glucose in the CD group and the HFD group after 10-week high fat diet before STZ injection. C-D: Insulin resistance test assessed by intraperitoneal glucose tolerance test and HOMA-IR. Black circle = the CD group, white triangle = the HFD group. E-G: TG/TC, Scr /BUN, ALT/ AST in the CD group and the HFD group after 10-week high-fat diet before STZ injection. Data are presented as mean ± SD (n = 12). *p<0.05, compared to CD group. CD, chow diet group; HFD, high-fat diet group. IPGTT, intraperitoneal glucose tolerance test; TC, total cholesterol; TG, total triglycerides; Scr, serum creatinine; BUN, blood urea nitrogen; ALT, alanine transaminase; AST, aspartate transaminase.

## Slide 2
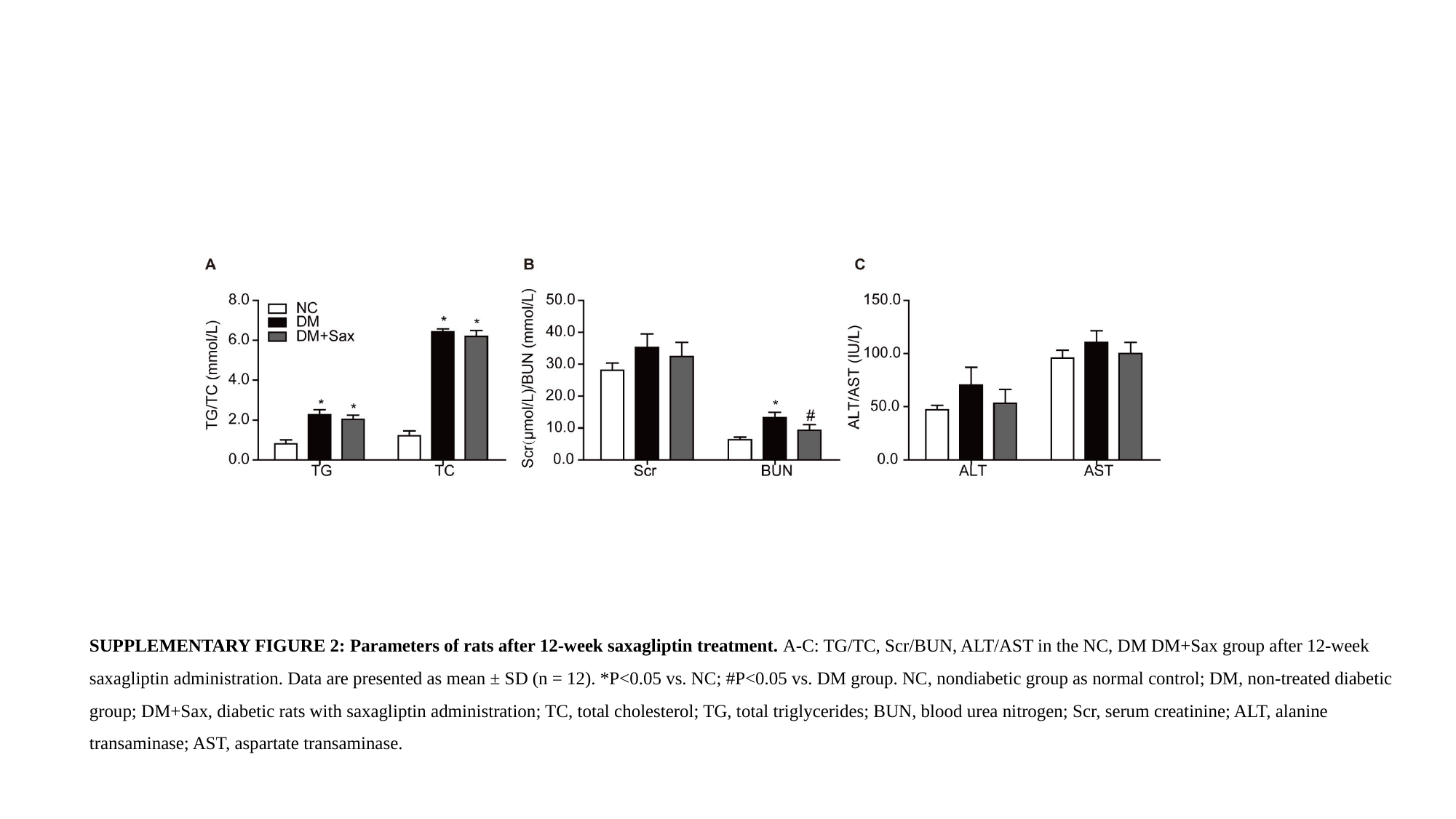

SUPPLEMENTARY FIGURE 2: Parameters of rats after 12-week saxagliptin treatment. A-C: TG/TC, Scr/BUN, ALT/AST in the NC, DM DM+Sax group after 12-week saxagliptin administration. Data are presented as mean ± SD (n = 12). *P<0.05 vs. NC; #P<0.05 vs. DM group. NC, nondiabetic group as normal control; DM, non-treated diabetic group; DM+Sax, diabetic rats with saxagliptin administration; TC, total cholesterol; TG, total triglycerides; BUN, blood urea nitrogen; Scr, serum creatinine; ALT, alanine transaminase; AST, aspartate transaminase.
